# Effects of Vitamin D3 Supplementation and Resistance Training on 25-Hydroxyvitamin D Status and Functional Performance of Older Adults: A Randomized Placebo-Controlled Trial

**DOI:** 10.3390/nu14010086

**Published:** 2021-12-26

**Authors:** Rudolf Aschauer, Sandra Unterberger, Patrick A. Zöhrer, Agnes Draxler, Bernhard Franzke, Eva-Maria Strasser, Karl-Heinz Wagner, Barbara Wessner

**Affiliations:** 1 Research Platform Active Ageing, University of Vienna, 1090 Vienna, Austria; rudolf.aschauer@univie.ac.at (R.A.); sandra.unterberger@univie.ac.at (S.U.); patrick.zoehrer@univie.ac.at (P.A.Z.); agnes.draxler@univie.ac.at (A.D.); bernhard.franzke@univie.ac.at (B.F.); karl-heinz.wagner@univie.ac.at (K.-H.W.); 2 Centre for Sport Science and University Sports, University of Vienna, 1150 Vienna, Austria; 3 Department of Nutritional Sciences, University of Vienna, 1090 Vienna, Austria; 4 Karl Landsteiner Institute for Remobilization and Functional Health/Institute for Physical Medicine and Rehabilitation, Kaiser Franz Joseph Hospital, Social Medical Center South, 1100 Vienna, Austria; eva-maria.strasser@wienkav.at

**Keywords:** vitamin D supplementation, 25-hydroxy-vitamin D, resistance training, physical fitness, mobility, older adults

## Abstract

Vitamin D status is associated with muscle strength and performance in older adults. To examine the additive effects of vitamin D3 supplementation during resistance training, 100 seniors (65–85 years) participated in a 16-week intervention. Besides a daily dose of 400 mg of calcium, participants received either 800 IU vitamin D3 per day (VDD), 50,000 IU vitamin D3 per month (VDM) or nothing (CON). After the initial loading phase of four weeks, all groups started a 10-week resistance training program. Assessments of 25-hydroxyvitamin D (25(OH)D) status, muscle strength endurance (30-s chair stand and arm curl tests), aerobic capacity (6-min walk test) and functional mobility (gait speed and timed up and go test) were undertaken at baseline, after four weeks and at the end of the study. 25(OH)D status significantly improved in VDD and VDM, but not in CON (time x group: *p* = 0.021), as 15.2% of CON, 40.0% of VDD and 61.1% of VDM reached vitamin D sufficiency (>30 ng/mL; *p* = 0.004). Chair stand test, arm curl test, 6-min walk test, gait speed and timed up and go test improved over the whole intervention period (*p* < 0.05), however only chair stand and arm curl test were selectively affected by resistance training (*p* < 0.001). Neither muscle strength endurance, nor functional mobility or aerobic capacity were modulated by vitamin D supplementation. Therefore, the mere amelioration of 25(OH)D status of older adults does not lead to an additive effect on muscular performance during RT.

## 1. Introduction

It is well known that ageing leads to a loss of strength and muscle mass as well as a decreased neuromuscular power [1,2]. As a result, the physiological degeneration of the musculoskeletal system causes several impacts on the life of the older adults such as an increased difficulty performing activities of daily living and recurrent risk of sudden falls and even death [3,4,5]. To tackle the above mentioned physical decline, it is widely accepted that resistance training (RT) represents an effective measure to reduce age-associated changes in the musculoskeletal system and different meta-analyses have conclusively proven the beneficial effects of weight-bearing exercises [6,7].

Aside from RT, vitamin D3 seems to play an important role in preventing age-related deteriorations. Once either absorbed from diet or produced by the skin through ultraviolet radiation, vitamin D3 is converted into its bioactive form 1,25-dihydroxyvitamin D3 via hydroxylations in liver and kidneys [8]. It has been shown that 1,25-dihydroxyvitamin D3 affects proliferation and differentiation of muscle cells [9,10]. Moreover, 25-hdroxyvitamin D (25(OH)D) status, which includes 25(OH)D3 and 25(OH)D2, has been repeatedly related to muscle strength [11,12,13] and physical performance outcomes important for activities of daily living such as standing up from a chair or gait speed [14,15,16]. Mechanistically, several pathways might be involved in the supportive role of vitamin D3 in restoring, maintaining or improving muscle function, ranging from the regulation of calcium homeostasis important for muscle contractions via increasing the function of mitochondria to directly affecting anabolic and catabolic pathways in skeletal muscle cells [17,18,19].

However, vitamin D supplementation with the aim to increase muscle mass and function in older adults is still under debate, since various studies have shown heterogeneous results which might be caused by different subject characteristics including baseline 25(OH)D and health status, varying vitamin D supplements (vitamin D2 and D3), dosages and supplementation regimes [20]. Even though 800 IU of vitamin D2 or D3 per day is considered the recommended dose for older people in many countries, it is assumed that higher amounts might be required to reach a sufficient 25(OH)D status of above 30 ng/mL and the associated benefits on the muscular system [21,22]. Beyond that, it is questionable whether vitamin D supplementation alone could be effective without simultaneously challenging the musculoskeletal system (i.e., by RT) as this has been shown to be important for other anabolic acting substances such as protein or testosterone [23,24,25].

Considering these issues, we hypothesized that any beneficial effects of RT would be increased when 25(OH)D status is elevated by supplementation of vitamin D3. Therefore, the aim of the current study was to evaluate whether the effects of a progressive RT on physical performance parameters would be modulated by two different vitamin D supplementation regimes in community-dwelling older adults with low to moderate baseline vitamin D levels.

## 2. Materials and Methods

### 2.1. Experimental Design

**Figure 1:**
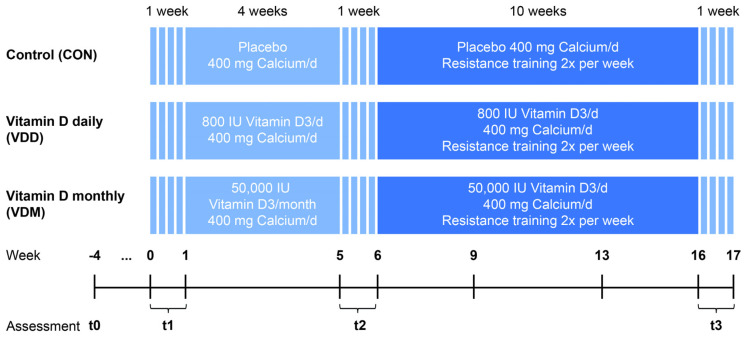
Study time line.

In this study, a randomized placebo-controlled double-blind design was used. Subject recruitment started in January 2019 involving a pre-assessment of inclusion and exclusion criteria (t0), while the main study lasted from mid-February to the middle of July 2019. The subjects were randomly allocated to three nearly even groups, the control group (CON), the vitamin D3 daily group (VDD) and the vitamin D3 monthly group (VDM). The study period was separated into two phases, a four-week vitamin D3 loading phase followed by a ten-week combined training and supplementation phase. Physical performance assessments were conducted and blood samples were taken at the beginning of the main intervention (t1), after the supplementation only phase (t2) and after the exercise plus supplementation phase (t3) (Figure 1). All tests and examinations were conducted at the Centre for Sports Science and University Sport of the University of Vienna, Austria (latitude: 48°12′).

### 2.2. Participants

Participant recruitment took place via newspaper, radio and online advertisements. For inclusion, participants needed to have an age between 65 and 85 years, an independent lifestyle (not living in a retirement home, not receiving aid by a home carer) and a Mini Mental State Examination (MMSE) of >23. Exclusion criteria were defined as having a 25(OH)D level > 30 ng/mL at t0, the need for any walking aid, chronic illness that would contradict a sports training therapy, severe cardiovascular disease (decompensated chronic cardiac insufficiency, extreme or symptomatic aortic stenosis, instable angina pectoris, untreated arterial hypertension, cardiac arrhythmia), diabetic retinopathy, osteoporosis or osteopenia with vitamin D and/or calcium substitution, kidney disease, kidney stones, disorder of the parathormone level, intake of cardiac glycosides, intake of diuretics (thiazides), disturbance of the calcium levels, a frailty index of three and higher, regular intake of cortisone or antibiotics (last six months), regular resistance exercise (>1x/week) in the last six months before study inclusion and missing declaration consent. To take part in this study, all inclusion criteria had to be met, while the presence of at least one exclusion criteria led to exclusion from the study. All subjects gave their informed consent for inclusion before they participated in the study. The study was conducted in accordance with the Declaration of Helsinki, and the protocol was approved by the Ethics Committee of the University of Vienna (Reference Number: 00390) and registered at https://clinicaltrials.gov (accessed on 5 December 2021) (NCT04341818).

### 2.3. Interventions

The whole intervention period consisted of two parts. The first phase, where the participants supplemented vitamin D3 or placebo but did not perform any additional exercise, lasted for four weeks. The second phase, where, alongside the vitamin D3 or placebo supplementation, a progressive RT was conducted, lasted for ten weeks.

#### 2.3.1. Nutrient Supplementation

Participants were allocated to CON, VDD or VDM groups. The CON group received a placebo in form of 400 mg of calcium each day (Mamisch GmbH Prorenal, Ludwigshafen, Germany). The VDD group took 400 mg of calcium plus 800 IU of vitamin D3 (Vitactiv Natural Nutrition, FeelGood Shop BV, Venlo, The Netherlands) on a daily basis, and the VDM group were set to 400 mg of calcium each day plus once every four weeks a dose of 50,000 IU vitamin D3 (Gall-Pharma GmbH, Judenburg, Austria). The vitamin D3 dose of 50,000 IU was provided in the laboratory during the assessments every four weeks (at t1, t2, weeks 9 and 13). All other supplements were handed out in 28-day pill boxes which provided the subjects with their daily intake doses. Returned pill boxes were controlled for supplements which had not been taken. Furthermore, participants were instructed to take the supplements together with a meal, always at the same time of the day.

#### 2.3.2. Resistance Training

The training program was based on the guidelines of the American College of Sports Medicine and the American Heart Association [26]. It lasted for ten weeks and was performed twice a week with a duration from 60 to 90 min for each session, adding up to a total of 20 possible sessions during the whole exercise intervention period. Week 1 was used for familiarization with the different exercises performed on two days and conducting two sets of 10 to 20 repetitions per exercise, whereas the third session (week 2) included all eight exercises, which were executed 8 to 12 times. During the fourth session, the five-repetition maximum (5RM) was evaluated according to Amarante do Nascimento et al. [27] and Wood et al. [28] in order to estimate the one-repetition maximum (1RM) using the reference table from Haff and Triplett [29]. The intensity of the rowing exercise was based on the results of the latissimus pulldown. Free weights and bodyweight exercises were not included in the 5RM assessments, but instead trainers and subjects determined the intensity together, based on the subjects’ daily condition. From week 3 to week 10, the participants trained based on their individual capacity at about 60–80% of their 1RM. The number of sets was raised to three and intensity increased when the participants were able to perform more than 12 repetitions.

The training sessions were conducted in carefully selected fitness centers providing similar training devices and conditions and included machine-guided exercises (leg press/leg curl/latissimus pulldown/rowing/chest press), free weight exercises (goblet box squat/dumbbell shoulder press) and one bodyweight exercise (front plank with alternating single leg raise). The used machines were part of the Technogym Selection Line 700 and 900 (Technogym S.p.A., Cesena, Italy). Training groups consisted of a maximum of five participants and were guided by qualified and trained persons (sport scientists). At the beginning of each training session, participants started with a five-minute warm up on a cross trainer or a bicycle followed by mobilization exercises of the loaded joints. The resistance exercises were executed pairwise in four supersets with eight exercises. Participants were instructed to execute each concentric phase of a repetition as fast as possible, while the eccentric phase was defined by a slower movement of two to three seconds. After the training session, participants performed a cool down. A more detailed exercise description can be found in Supplementary File 1 (Table S1).

### 2.4. Outcomes

Outcome assessment consisted of anthropometric and physical activity (steps per day) measures, which were only used for baseline analyses, the 30-s chair stand test and the 25(OH)D status as the primary outcomes, and the arm curl test, the timed up and go test, the gait speed and the six-minute walk test as secondary outcomes.

#### 2.4.1. Vitamin D Status

Venous blood samples were taken in the morning between 06:30 and 09:00 after an overnight fast. Z Serum Sep Clot Activator collection tubes (Vacuette, Greiner bio-one, Kremsmünster, Austria) were used to obtain about 8 mL of venous blood. After a clotting time of at least 30 min, the tubes were centrifuged (3000 rpm, 10 min, room temperature) and the supernatant (serum) samples were sent to a routine laboratory (Dr. Claudia Vidotto, 1230 Vienna, Austria) for subsequent analyses of the 25(OH)D status using the Access 25(OH) Vitamin D Total assay (Beckman Coulter Austria, Vienna) which detects both 25(OH)D2 and 25(OH)D3.

#### 2.4.2. 30-s Chair Stand Test

Before the test, a standardized warm up combining mobilization and static stretching exercises was performed. According to the guidelines by Rikli and Jones [30] the 30-s chair stand test was conducted to analyze the participants’ lower limb function. While sitting on a platform of 43 cm height, the knees should be bent in an angle of about 90°. The subjects were told to stand up to an upright position and sit down in a controlled manner as many times as possible during a period of 30 s. When standing up and sitting down again, bouncing should be avoided. The tester gave verbal encouragement and counted the number of repetitions. The last repetition was taken into consideration when at least half of the movement was completed.

#### 2.4.3. 30-s Arm Curl Test

The arm curl test over 30 s was performed, again oriented on the Senior Fitness Manual by Rikli and Jones [30]. Both arms were tested, starting with the right one. Men used a 3.6 kg dumbbell and women a 2.3 kg dumbbell. Participants were instructed and allowed to familiarize themselves with the test without any additional weight. For the test, participants were encouraged to complete as many correct arm curls as possible within a time limit of 30 s while sitting on a chair. The number of repetitions correctly completed were counted and used for further analyses. The last repetition counted when more than 50% were completed.

#### 2.4.4. Timed up and Go Test

According to the guidelines by Podsiadlo and Richardson [31] participants started the test by sitting on a chair with arms on the thighs. After a verbal starting signal, participants stood up and walked as fast as possible along a track of three meters, turned around a cone and went back to sit down on the chair. The test was counted as finished when the participants’ buttocks touched the chair. Time was measured to the nearest 0.01 s with a stopwatch and the best of two trials was used for statistical analyses as recommended by Collado-Mateo et al. [32].

#### 2.4.5. Gait Speed

For this test, six light beams (Brower Timing System, Draper, UT, USA) were set up pairwise across each other at zero, four and six meters. The starting- and end-point were marked with a cone two meters before the first pair of light barriers and two meters after the last pair. Therefore, participants covered an actual distance of ten meters, whereby time was measured for covering a distance of six meters. The subjects were asked to walk as fast as possible and not to decelerate before the finish line. From two trials interspersed by a break of two minutes, the faster trial was used for further analyses. Gait speed was calculated by transforming walking time for 6 m (to the nearest 0.01 s) to gait speed in m/s [33].

#### 2.4.6. Six-Minute Walk Test

For the evaluation of aerobic endurance, the 6-min walk test was applied according to Rikli and Jones [30]. Briefly, subjects tried to walk (not run) as fast and far as possible on a 30-m track with marks at every five meters. Participants were informed over the elapsed time every minute. The tester reported every finished round of 30 m. A fixed measuring tape parallel to the track provided information about the finally covered distance to the nearest 1.0 m.

#### 2.4.7. Anthropometry

Anthropometric measures consisted of height and weight measurements, as well as the subsequent calculation of the subjects’ body mass index (BMI). Weight and height were evaluated with a medical scale (model 877, seca GmbH & Co. KG, Hamburg, Germany) attached to a stadiometer (model 217, seca GmbH & Co. KG, Hamburg, Germany) to the nearest 0.1 kg and 0.1 cm, respectively. BMI was calculated as weight in kg divided by height in m squared.

#### 2.4.8. Step Counts

The ActiGraph GT1M (ActiGraph, LLC, Pensacola, FL, USA) was used for counting steps and ActiLife v5.10.0 software (ActiGraph, LLC) for evaluation [34]. Participants had to wear the accelerometer for at least 4 days a week, with a minimum number of 3 days during the week and 1 day of the weekend. The minimum wear time per day was set to 10 h. A minimum of 5% of non-zero epochs per hour was required. Furthermore, counts below 0 and over 30,000 were considered as erroneous measurements and were not included in the analyses.

### 2.5. Statistics

#### 2.5.1. Sample Size Determination

The number of participants was calculated using the G*Power software, version 3.1.9.2 [35]. Based on an assumed effect size f of 0.15, the alpha level set to 0.05 and the power (1–β) to 0.80, the a priori power analyses assuming an ANOVA with three groups and three measurement time points revealed a total sample size of 93 participants. A previous training study from our laboratory showed a drop-out rate of about 30% [36], therefore we aimed to recruit a total of 121 participants.

#### 2.5.2. Randomization and Blinding

Participants that met the criteria were randomly allocated to one of the three intervention groups using an academic randomization tool (https://randomizer.at/, Institute for Medical Informatics, Statistics and Documentation, Medical University of Graz, Graz, Austria, accessed on 5 December 2021). As physical performance parameters might have been influenced by age, sex and vitamin D status, subjects were stratified by age (65 to 69.9 years; 70 to 74.9 years; 75 to 79.9 years; 80 to <85 years), sex (female; male) and baseline vitamin D level (<20 ng/mL; 20–30 ng/mL) in order to reach similar conditions at baseline. Working staff as well as participants were blinded with regard to supplementation group allocation as none of the testers or trainers were involved in preparing the pill boxes forwarded to the participants.

#### 2.5.3. Statistical Analyses

The evaluation was based on an intention-to-treat analysis, specifically meaning that participants were included regardless of training compliance. For data clearance, all entries to the statistical software (IBM SPSS Statistics 25.0) were controlled twice by two independent researchers. Furthermore, extreme values for each parameter were checked again for transcription errors as well as for physiological plausibility and only excluded if a measurement error or problem was ascertained. Vitamin D status at the beginning was assessed twice at t0 and t1. In order to obtain reliable baseline values, an average of these two measurements was used for further analysis.

Normal distribution of metric variables was tested by visual inspection of the histograms as well as by the Shapiro–Wilk test. An appropriate transformation for all affected parameters was performed, if the normal distribution was violated (square root transformation for 30-s chair stand and 6-min walk tests, log-transformation for 30-s arm curl test and step counts, inverse transformation for timed up and go test, BMI, and body mass).

Differences between groups at baseline were tested by one-way ANOVA for metric variables or chi-squared test for categorical measures. For assessment of time (within subject factor), group (between subject factor) and time x group interaction effects, a two-way mixed ANOVA with Bonferroni-corrected post hoc analyses was used. Homogeneity of variances was tested either by the Levene test and/or by Mauchly’s test of sphericity. If the latter was violated, Greenhouse Geisser-corrected values were shown. Simple main effects for group and time were analyzed in the case of a significant group x time interaction. Non-significant interactions were interpreted by the main effects for time and group. In order to compare the rate of changes between study phase 1 and 2, differences were calculated (t2 − t1 and t3 − t2) and further examined by paired sampled *t*-tests.

Since transformed variables were used for various analyses, data in tables are stated as medians (25th–75th percentiles) in order to provide more information on the exact data distribution. Furthermore, exact *p*-values, test statistics and effect sizes (partial η^2^ or Cohen’s d) are provided.

## 3. Results

### 3.1. Participant Flow and Baseline Characteristics

**Figure 2:**
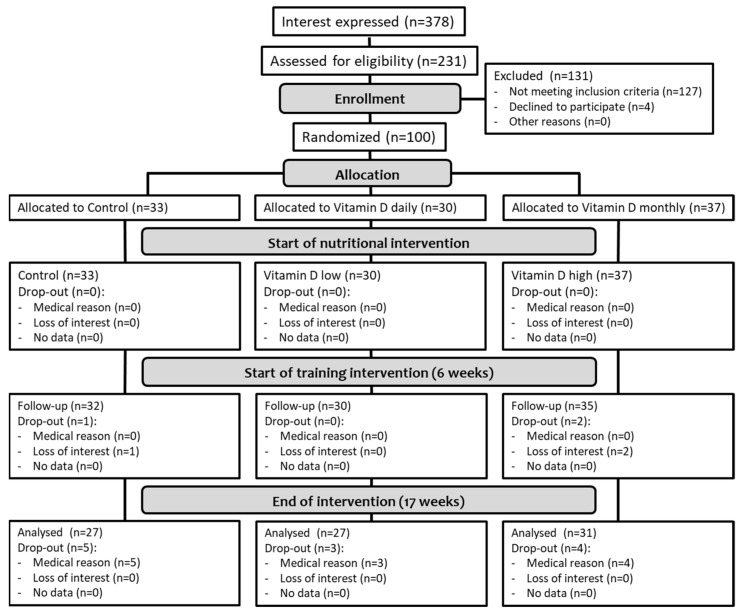
Participant flow chart.

From 231 participants expressing their interest in participating in the study, only 100 (43.3%) could be included (Figure 2). The main reasons for exclusion were 25(OH)D level > 30 ng/mL (39 males, 55 females) followed by medical issues such as untreated hypertension, osteoporosis or calcium prescription. Based on the block size used for randomization, the distribution to the groups was not exactly even, with 33 participants allocated to the CON (33.0%), 30 to the VDD (33.0%) and 37 to the VDM (37.0%). Three participants quit the study during the nutritional intervention phase from t1 to t2 as they lost interest in participating in the study (3.0%), while a further 12 participants (12.0%) had to stop their participation due to medical reasons during the combined supplementation and training phase from t2 to t3, resulting in an overall drop-out rate of 15% (Figure 1). The frequency of drop-outs was similar between groups (χ^2^(2) = 0.893; *p* = 0.640). Drop-outs (*n* = 15) did not differ from those finishing the study with respect to age, body mass, 30-s chair stand and arm curl tests, gait speed, timed up and go, 6-min walk test and step counts at t1. 

Compliance for vitamin D3 supplementation was 100% in the VDM and 99% in the VDD. From t2 to t3 participants completed 19 (17–20) out of the 20 possible training sessions with no differences between groups (CON: 19 (17–20); VDD: 19 (18–20); VDM: 19 (17–20), *p* = 0.966).

At baseline, no significant group differences could be observed for sex distribution, age, height, weight, BMI, physical activity (steps per day), and 25(OH)D status (Table 1).

### 3.2. 25(OH)D Status

There was a statistically significant interaction between groups and time points for 25(OH)D level (F(3.186, 130.611) = 3.278, *p* = 0.021; partial η^2^ = 0.074). Further analysis of the simple main effect for groups at the different time points revealed no differences at t1 (F(2, 97) = 0.838, *p* = 0.436, partial η^2^ = 0.017) and t2 (F(2, 94) = 2.215, *p* = 0.115, partial η^2^ = 0.045), while differences in 25(OH)D status were observed at the end of the intervention at t3 (F(2, 82) = 5.696, *p* = 0.005, partial η^2^ = 0.122). At this time point, Bonferroni-corrected post hoc analyses showed higher values for VDM (32.7 (28.9–35.7) ng/mL) compared to the CON (23.7 (20.4–28.1) ng/mL) (*p* = 0.003). VDD (29.0 (23.8–33.3) ng/mL) levels were not different to CON (*p* = 0.362) or VDM (*p* = 0.136). Interestingly, simple main effects for time revealed a significant increase in 25(OH)D in all groups: CON (F(1.274, 33.117) = 4.285, *p* = 0.038, partial η^2^ = 0.141), VDD (F(2, 52) = 9.363, *p* < 0.001, partial η^2^ = 0.265), and VDM F(1.648, 49.431) = 32.597, *p* < 0.001, partial η^2^ = 0.521), but Bonferroni-corrected post hoc analyses showed significant improvements in VDM and VDD from t1 to t3 (VDM: *p* < 0.001; VDD: *p* = 0.003) and from t2 to t3 (VDM: *p* < 0.001; VDD: *p* = 0.012), but not in CON. Data are summarized in Table 2.

We further classified participants based on their individual 25(OH)D level as deficient (<20 ng/mL), insufficient (20 to 30 ng/mL) or sufficient (>30 ng/mL) [21]. The superiority of the VDM supplementation regime was also seen in the frequency of subjects that shifted to a sufficiently high 25(OH)D level at t3. In CON, only 5 subjects (15.2 %), in VDD 12 subjects (40.0%) and in the VDM 22 subjects (61.1%) reached vitamin D sufficiency (χ^2^(4) = 15.549; *p* = 0.004).

### 3.3. Primary Outcome (30-s Chair Stand Test)

The two-way mixed ANOVA did not reveal a significant group x time interaction effect (F(3.468, 140.461) = 0.291, *p* = 0.859, partial η^2^ = 0.007). Irrespective of group allocation, the number of repetitions in the 30-s chair stand test increased over time (F(1.734, 140.461 = 84.208, *p* < 0.001, partial η^2^ = 0.510), whereby the Bonferroni-corrected post hoc tests confirmed improvements from t1 to t2 (*p* < 0.001) and t2 to t3 (*p* < 0.001). Of note, the increase was significantly higher in the RT intervention phase from t2 to t3 as compared to the vitamin D3 loading phase from t1 to t2 (T(83) = 2.023, *p* = 0.046, d = 0.220) (Table 2).

### 3.4. Secondary Outcomes (Physical Performance)

The two-way mixed ANOVA did not acknowledge any significant group x time interaction for 30-s arm curl test, timed up and go, gait speed, and 6-min walk test (*p* > 0.05). Similarly to 30-s chair stand test, performance in the arm curl test of the dominant as well as of the non-dominant side increased over time (dominant: F(2, 160 = 106.878, *p* < 0.001, partial η^2^ = 0.572; non-dominant: F(2, 158 = 113.733, *p*< 0.001, partial η^2^ = 0.590), whereby the increase was significantly higher in the second phase where RT was added to the vitamin D3 supplementation protocols (dominant: T(82) = 4.599, *p* < 0.001, d = 0.505; non-dominant: T(81) = 4.243, *p* < 0.001, d = 0.469).

Further significant main effects for time were observed for gait speed (F(2, 164) = 4.220, *p* = 0.016, partial η^2^ = 0.049), the timed up and go test (F(1.849, 151.656) = 5.177, *p* = 0.008, partial η^2^ = 0.059), and the 6-min walk test (F(2, 162) = 4.454, *p* = 0.013; partial η^2^ = 0.052). Bonferroni-corrected post hoc analyses revealed significant improvements over the whole intervention period from t1 to t3 for gait speed (*p* = 0.022), timed up and go test (*p* = 0.021), and 6-min walk test (*p* = 0.040). Only for the 6-min walk test was the increase already evident between t1 and t2 (*p* = 0.045), with no further change between t2 and t3 (*p* = 1.000). These observations correspond to the results of the paired sample *t*-tests which did not reveal any significant differences between the two intervention phases (gait speed: *p* = 0.343, d = 0.102; timed up and go test: *p* = 0.404, d = 0.091; 6-min walk test: *p* = 0.194, d = 0.143).

### 3.5. Covariates (Body Mass, Physical Activity)

In order to assess whether the results could have been affected by changes in physical activity, body mass or BMI, the impact of the intervention on those parameters was determined. 

For body mass and BMI the two-way mixed ANOVA did not reveal any time × group interaction effect (body mass: F(3.416, 140.075) = 0.407, *p* = 0.774, partial η^2^ = 0.059; BMI: F(3.483, 142.793) = 0.192, *p* = 0.924, partial η^2^ = 0.005). As for the main effects for time (body mass: F(1.708, 140.075) = 8.591, *p* = 0.001, partial η^2^ = 0.095), BMI: F(1.741, 142.793) = 5.084, *p* = 0.010, partial η^2^= 0.058) all groups slightly lost weight from t1 to t3 (80.3 (68.4–90.7) kg vs. 79.8 (68.0–90.7) kg, *p* = 0.002) as determined by Bonferroni-corrected post hoc analyses. 

For physical activity as assessed by daily step counts, a small, but significant, time x group interaction effect was revealed (F(4, 116) = 3.139, *p* = 0.017, partial η^2^ = 0.098). Therefore, simple main effects were calculated separately for the groups. However, none of the groups changed their daily step counts significantly (CON: F(2, 38) = 2.259, *p* = 0.118, partial η^2^ = 0.106; VDD: F(2, 40) = 2.352, *p* = 0.108, partial η2 = 0.105; VDM: F(2, 38) = 3.200, *p* = 0.052, partial η^2^ = 0.144). Medians (25th–75th percentiles) in CON were 7022 (5808–9855), 6866 (5903–8021), and 5982 (5277–9540) steps/day, in VDD 7143 (5538–9881), 8029 (5858–9139), and 7860 (5909–10317) steps/day and in VDM 6927 (4430–9403), 7529 (5439–10052), and 7601 (5716–11808) steps/day at t1, t2 and t3, respectively. 

## 4. Discussion

The aim of the current study was to investigate whether vitamin D3 supplementation would modulate the effects of a progressive RT on functional performance in community-dwelling older subjects with vitamin D insufficiency. While VDM especially had the potential to increase the 25(OH)D status to levels above 30 ng/mL, none of the tested functional performance parameters were selectively affected by vitamin D3 supplementation.

25(OH)D status continuously improved over time with both supplementation modalities reaching significance at week 16 (t3), whereby the increase in 25(OH)D levels reached 26% and 40% for VDD and VDM, respectively. Although 25(OH)D levels did not differ between VDD and VDM at this time point, a higher proportion of subjects in VDM reached vitamin D sufficiency (40% versus 61%) suggesting a slight superiority of VDM. It has to be noted that the total supplemented dose summed up to 89,600 IU in the VDD group administered at the recommended dose of 800 IU/d for this age group [21]. In contrast, the VDM group received 200,000 IU vitamin D3 supplemented as 50,000 IU per month, which would correspond to an amount of 1785 IU/d. A slightly higher improvement of 61% has been shown previously with a smaller total amount but a higher daily dose of 2800 IU/d vitamin D3 for eight weeks in a comparable population [37]. Similar data are provided in a dose–response study where 25(OH)D concentrations increased with dose and were highest at 50,000 IU/week, followed by 4000 IU/d, 2000 IU/d and 800 IU/day with no differences between the latter two [38]. With respect to the supplementation regime, there seems to be no difference in affecting 25(OH)D status, when the same total dose was administered daily, weekly or monthly (1000 IU/d, 7000 IU/week or 30,000 IU/month) over a period of three months. Furthermore, equal safety profiles were obtained [39]. Taken together, these data seem to confirm that doses above 800 IU/d might be required to efficiently improve a low vitamin D status, but daily, weekly or monthly administrations provide equal efficacy. 

Interestingly, some studies used oil drops instead of pills, which raises the question if the form of delivery would play a role. When comparing oil drops and tablets in a nearly equal dose (tablets: 1600 IU per day; oil drops: 1500 IU per day) for 12 months, no differences in 25(OH)D improvements have been found [40]. Besides preparation, different forms of vitamin D are available for supplementation such as vitamin D3, vitamin D2 or its metabolites 25(OH)D or 1,25-dihdroxyvitamin D [41]. In the current study, vitamin D3 was used, which has been shown to be more efficacious at raising serum 25(OH)D concentrations than vitamin D2 [42]. However, direct supplementation of 25(OH)D might even be superior in increasing vitamin D status [43,44].

It has been frequently shown that low 25(OH)D status is associated with poor physical performance [11,13,45,46]. Furthermore, treatment with 0.5 µg alphacalcidiol over 6 months seems to improve muscle strength and walking distance over 2 min only in vitamin-D-deficient older women [47]. This was confirmed in a meta-analysis which revealed that vitamin D supplementation alone does not have a significant effect on muscle strength in adults with 25(OH)D levels above 10 ng/mL, whereby a limited number of studies demonstrate an increase in proximal muscle strength in adults with vitamin D deficiency [48]. Therefore, we hypothesized that improving the 25(OH)D status in older adults would exert an additive effect during RT. Unfortunately, we were not able to detect any differences between CON, VDD and VDM related to functional performance as assessed by 30-s chair stand and arm curl tests, timed up and go, gait speed, and 6-min walk test. Further analyses revealed that there was no correlation between increases in 25(OH)D levels and changes in physical performance (data not shown). This is in line with intervention studies, which showed no additive effect of vitamin D3 intake during different forms of exercise on physical functioning, muscle strength or aerobic capacity [49,50,51]. Nonetheless, a meta-analysis including only three studies with moderate quality revealed a slight beneficial effect on lower limb muscle strength, but none on functional performance as assessed by timed up and go test [52]. Therefore, it is difficult to draw a firm conclusion and it cannot be ruled out that other parameters such as muscle quality, fiber type morphology or muscular hypertrophy signaling might have been improved regardless of changes in physical performance [51]. 

A possible important factor for performance improvements might be the baseline 25(OH)D status and more specifically the level to which it could be enhanced. Bischoff-Ferrari et al. [11] have shown that for optimal lower-extremity function, 25(OH)D concentrations as high as the upper end of the reference range (36–40 ng/mL) appear to be advantageous. In the current study, only two participants had 25(OH)D concentrations above 36 ng/mL at the beginning of the RT (t2) and 13 participants reached that concentration at t3 (CON: 3 (11.1%), VDD: 3 (11.1%), VDM: 7 (22.6%)). 

The effectiveness of the RT was estimated by comparing the changes in functional performance parameters between phase 1 and 2, which should show a steeper increase (or decrease for timed up and go test) in phase 2. The tested parameters, strength (endurance) of the lower and upper body as estimated by the functional 30-s chair stand and arm curl tests [53,54], showed the expected differences between phase 1 and 2, whereas gait speed, timed up and go (functional mobility) and the 6-min walk test (aerobic performance) were not affected by the applied RT. The significant improvements in chair stand and arm curl tests as well as the missing changes in the 6-min walk test due to RT coincide with previous studies [55,56,57]. Furthermore, it has been suggested that fast-intended-velocity RT may elicit greater improvements in functional capacity when compared to moderate-velocity RT [58]. Therefore, the instructions given to the participants in this study included a low to moderate concentric velocity (≥2 s for each eccentric phase) and a maximal velocity (as fast as possible) in the concentric phase. Nevertheless, we did not observe any amelioration in timed up and go test and gait speed, which parallels the results of a recent meta-analysis [59]. It has to be mentioned that the RT phase lasted 10 weeks including familiarization, training and progression, which might have been too short to reach greater improvements [60]. 

Besides the RT-specific increase in chair stand and arm curl performance, it is noteworthy that also gait speed, timed up and go, and 6-min walk test improved from t1 to t3, and the 6-min walk test from t1 to t2. One reason for these general improvements might have been that subjects were cautious at the beginning and gained confidence after the first trials, hence improving significantly over time. Although a good test–retest reliability has been ascertained for all used tests [30,61], special care was taken to familiarize the participants with each of the tests to minimize potential habituation effects. The impact on the overall interpretation of the study should be small as all groups were treated equally. 

Finally, potential limitations of this study have to be mentioned. Although the study started after the Christmas holidays in late winter, the rather long duration of the intervention with a total length of 17 weeks per person and the limited capacity to test and train the participants led to the finalization of the study in July. In 2019, the daily amount of sunshine hours increased from 5.5 to 9.2 h/d within the study period. Although the seasonal change might have influenced the vitamin D status, our data reveal that 25(OH)D levels of the CON group did not significantly increase during the second phase of the study. Therefore, seasonal changes might have played a minor role. Furthermore, we cannot rule out that individuals changed their lifestyle during the intervention. With respect to physical activity, it can be assumed that at least step counts were similar between groups and did not change over time, but it cannot be excluded that other activities not detected by accelerometers such as swimming or biking could have influenced the outcome. As sample size in intervention studies is considered to be critical, especially when small effects are to be detected, it has to be mentioned that with *n* = 85, the number of participants completing the study was slightly smaller than the anticipated 93 persons. However, this was mainly the result of the surprising observation that more than 100 elderly subjects, mainly women, could not be enrolled into the study due to high 25(OH)D levels at t0, indicating that in this age group a vitamin D3 supplementation prescribed by the general practitioner takes place routinely. Although only 15% were lost to follow-up, this could have affected the interpretation of our study. However, the effect size for the time x group interaction of the 30-s chair stand test was 0.007, indicating a small effect if any. Finally, this study was limited to independent subjects with a low 25(OH)D status, not taking any supplements. Therefore, the results of the study are only representative for the indicated target group.

## 5. Conclusions

In summary, the results of this study reveal that vitamin D supplementation for 16 weeks in the form of 800 IU per day (VDD) or four doses of 50,000 IU every four weeks (VDM) improved the 25(OH)D3 status of community-dwelling older adults with vitamin D insufficiency to a similar extent, with slightly more advantageous effects for the higher-dosed supplementation strategy. While ten weeks of RT improved lower and upper limb strength as functionally assessed by CST and ACT, no additive effect of either VDD or VDM was observed. While a sufficiently high level of vitamin D is important for several health aspects ranging from fracture and fall prevention, to cardiovascular disease risk or immunity, its direct association with muscle traits is still to be elucidated.

## Figures and Tables

**Table 1:** Baseline characteristics of participants.

	CON	VDD	VDM	*p*-Value	Partial η^2^
**Sex** (f/m)	10/23 (30.3% f)	10/20 (33.3% f)	13/24 (35.1% f)	0.911	
**Age** (years)	68.9 (67.0–72.9)	68.7 (66.9–74.8)	69.9 (67.0–74.3)	0.763	0.005
**Body mass** (kg)	77.4 (65.5–83.4)	85.8 (73.5–93.1)	80.3 (72.3–93.8)	0.123	0.042
**Height** (m)	1.73 (1.66–1.79)	1.73 (1.66–1.79)	1.73 (1.67–1.81)	0.932	0.001
**BMI** (kg/m²)	25.7 (23.0–27.5)	27.2 (25.1–30.7)	27.6 (24.4–30.0)	0.059	0.057
**25(OH)D** (ng/mL)	22.8 (18.2–26.6)	23.8 (20.1–28.1)	23.4 (18.6–26.4)	0.436	0.017
**Steps** (counts/d)	7022 (5590–9588)	7021 (4937–9353)	6610 (5208–9247)	0.482	0.016

Values are shown as medians (25th–75th percentiles); *n* = 100 unless for step counts (*n* = 94 due to invalid data); *p*-values refer to differences between groups (chi-square test, ANOVA). Abbreviations: CON (control group), VDD (vitamin D daily group), VDM (vitamin D monthly group), BMI (body mass index), f (female), m (male).

**Table 2:** Effects of vitamin D3 supplementation and RT on functional performance.

Parameter	Group	t1	t2	t3	Δ (t2 − t1)	Δ (t3 − t2)	Time	Group	Time × Group
**25(OH)D** (ng/mL), *n* = 85	CON	21.7 (17.3–27.0)	22.1 (17.3–21.3)	23.7 (20.4–28.1)	0.29 (−1.82–1.83)	1.32 (−1.69–6.35)	<0.001	0.022	0.021
	VDD	23.7 (18.9–28.1)	24.2 (21.3–26.7)	29.0 (23.8–33.3) **,°	1.26 (−1.42–3.49)	3.80 (−0.43–6.90)			
	VDM	23.4 (18.8–25.9)	26.0 (21.5–29.7)	32.7 (28.9–35.7) ***,°°°	3.11 (−0.04–4.84)	6.62 (3.60–10.91) ##			
**30-s chair stand test** (reps), *n* = 84	CON	13 (11–14)	14 (12–15)	15 (14–18)	1 (0–2)	1 (1–3)	<0.001	0.316	0.859
	VDD	13 (11–15)	14 (13–16)	16 (14–18)	1 (0–3)	1 (0–3)			
	VDM	12 (11–14)	13 (12–15)	16 (13–17)	1 (0–2)	2 (0–3)			
	total	12 (11–14)	14 (12–15) ***	15 (14–17) ***,°°°	1 (0–2)	1 (0–3) #			
**30-s arm curl test (dom)** (reps), *n* = 83	CON	18 (16–20)	20 (17–21)	22 (20–24)	1 (0–2)	3 (2–5)	<0.001	0.311	0.659
	VDD	19 (15–22)	20 (19–25)	23 (20–27)	1 (0–3)	3 (1–4)			
	VDM	19 (17–21)	19 (17–22)	22 (20–24)	1 (−1–2)	3 (1–6)			
	total	19 (16–21)	19 (17–21) ***	22 (20–24) ***,°°°	1 (0–2)	3 (1–5) ###			
**30-s arm curl test (non–dom)** (reps), *n* = 82	CON	17 (16–21)	18 (16–21)	21 (19–24)	0 (−1–2)	3 (1–5)	<0.001	0.213	0.327
	VDD	18 (15–21)	20 (18–24)	22 (21–25)	2 (0–3)	2 (1–5)			
	VDM	18 (16–20)	20 (17–21)	22 (20–24)	1 (0–3)	3 (1–5)			
	total	18 (16–21)	19 (17–21) ***	22 (20–24) ***,°°°	1 (0–3)	3 (1–5) ###			
**Timed up and go test** (s), *n* = 85	CON	4.72 (4.16–5.20)	4.71 (4.10–5.10)	4.45 (3.96–5.38)	−0.07 (−0.19–0.13)	−0.15 (−0.36–0.21)	0.008	0.916	0.470
	VDD	4.82 (4.24–5.34)	4.44 (3.99–5.30)	4.86 (4.03–5.18)	−0.25 (−0.46–0.07)	0.12 (−0.22–0.44)			
	VDM	4.45 (4.19–4.81)	4.57 (4.08–5.11)	4.41 (4.07–4.99)	−0.09 (−0.37–0.30)	−0.09 (−0.39–0.13)			
	total	4.66 (4.19–5.24)	4.57 (4.06–5.11)	4.50 (4.04–5.08) *	−0.09 (−0.38–0.14)	−0.08 (−0.35–0.25)			
**Gait speed** (m/s), *n* = 85	CON	1.54 (1.47–1.76)	1.69 (1.36–1.89)	1.67 (1.44–1.83)	0.04 (−0.02–0.16)	0.06 (−0.13–0.12)	0.016	0.823	0.320
	VDD	1.59 (1.37–1.83)	1.75 (1.45–1.87)	1.61 (1.36–1.88)	0.04 (−0.05–0.14)	−0.04 (−0.14–0.09)			
	VDM	1.66 (1.41–1.84)	1.63 (1.50–1.86)	1.65 (1.48–1.87)	0.02 (−0.08–0.12)	0.06 (−0.03–0.15)			
	total	1.61 (1.42–1.83)	1.68 (1.48–1.86)	1.65 (1.47–1.85) *	0.04 (−0.06–0.14)	0.03 (−0.12–0.13)			
**6-min walk test** (m), *n* = 84	CON	636 (549–674)	643 (570–692)	628 (581–687)	18.8 (−4.9–34.7)	6.7 (−19.8–15.3)	0.013	0.593	0.848
	VDD	610 (570–691)	618 (571–724)	623 (554–723)	1.3 (−17.8–30.4)	−1.9 (−23.8–24.6)			
	VDM	654 (605–697)	660 (627–716)	638 (604–713)	10.0 (−14.4–28.4)	−0.9 (−21.7–28.6)			
	total	641 (573–689)	648 (574–707) *	628 (584–718) *	9.9 (−10.6–29.29	−0.05 (−22.5–20.4)			

Values are shown as medians (25th–75th percentiles). *p*–values indicate the main effects for time, group and time x group interaction (two-way mixed ANOVA). Asterisks indicate significant differences to t1 and circles show differences to t2 (either separately for groups (25(OH)D) or for combined groups (total). Hashes show differences between deltas as calculated by paired *t*-tests; ***, °°°, ### (*p* < 0.001); **, ## (*p* < 0.01); *, °, # (*p* < 0.05). Abbreviations: RT (resistance training); CON (control group); VDD (vitamin D daily group); VDM (vitamin D monthly group); 25(OH)D (25-hydroxy vitamin D); dom (dominant arm); non-dom (non-dominant arm); reps (repetitions); t1 (baseline); t2 (start of RT); t3 (final assessment); t2 − t1 (change in phase 1 with supplementation only); t3 − t2 (change in phase 2 (supplementation plus RT).

## Data Availability

The data presented in this study are available on request from the corresponding author.

## Supplementary Materials

The following are available online at https://www.mdpi.com/article/10.3390/nu14010086/s1, Supplementary file 1: Training and exercise description, Table S1: exercise description.
