# Supplementary material for: Effects of Vitamin D3 Supplementation and Resistance Training on 25-Hydroxyvitamin D Status and Functional Performance of Older Adults: A Randomized Placebo-Controlled Trial
Source: Nutrients. 2021 Dec 26;14(1):86. doi: 10.3390/nu14010086 (PMC8746949; doi:10.3390/nu14010086)
Supplement: Supplementary file 1 [file nutrients-14-00086-s001.zip › nutrients-1519491-supplementary.pdf]

**Supplementary file. Training and exercise description**

Text S1: Training and exercise description

The resistance training was conducted twice a week for ten weeks. Each session lasted between 60 and 90 minutes. Exercises got performed in super set form. A more detailed exercise description can be seen below in the supplement figures. The pairs were A1/A2, B1/B2, C1/C2 and D1/D2. In the first two weeks participants got familiarized by executing two sets per exercise with 10-20 repetitions in the first week. From the third session onwards it was aimed to conduct 8-12 repetitions per exercise. Furthermore, in the first week only A1 to B2 got trained in the first session and C1 to D2 in the second session. The second session of week two was used to evaluate the participants 5RM. From week three until week ten, the subjects performed three sets with an intensity of 60 to 80% of their actual 1RM.

Table S1: *Exercise description*

<table>
<tr><th colspan="2">Lower body</th></tr>
<tr><td>M. gluteus maximus, medius, M. quadriceps femoris *</td><td>

**<u>A1: Goblet Box Squat</u>**<br>
*Starting position: sitting on the box with the kettlebell holding chest high; feet are positioned shoulder width, positioned forward/slightly outward*

- Move with a straight upper body upwards; knees should not move inside; try to exhale while moving up
- Move down again; meanwhile try to inhale; touch slightly the box with the buttock and repeat the movement

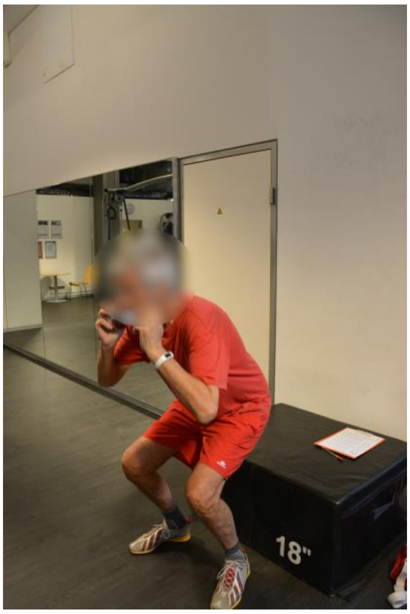


</td></tr>
<tr><td>M. biceps femoris, M. semitendinosus, M. semimebranosus, M. gastrocnemius</td><td>

**<u>A2: Leg Curl</u>**<br>
*Starting position: sitting on the machine, legs extended to an angle which still feels comfortable; the hands are holding the handles on the side*

- Pressing the machine with the legs down to an angle of about 90° or more; then going back up again in a controlled way; exhale while moving down, inhale while going up

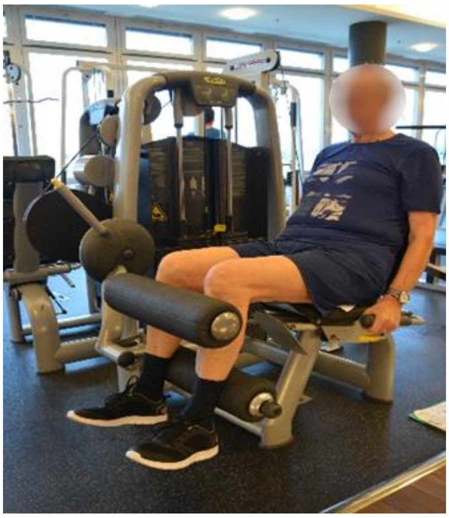

</td></tr>
</table>

| Upper body | |
|---|---|
| M. triceps brachii, M. deltoideus | **B1: Dumbbell shoulder press**<br>*Starting position: sitting with a straight upper body on a bench; upper arms are in prolonged position to the shoulders, while the hands are holding the dumbbells on head height*<br>o Press the dumbbells up until the elbows are extended<br>o Return to the starting position<br>o Exhale while pressing the weight up; inhale when moving down<br>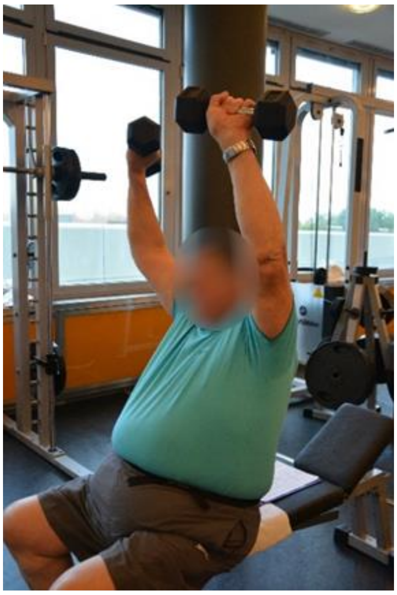 |
| M. latissimus dorsi, M. teres major, M. biceps brachii, M. brachialis | **B2: Latissimus pull**<br>*Starting position: sitting on the bench; thighs are press against the pad; arms are in extended position and hands are holding the bar*<br>o Pull the weight down until the bar reaches collarbone<br>o Let the weight move back up again in a controlled way<br>o Exhale while pulling the weight down; inhale when moving back up<br>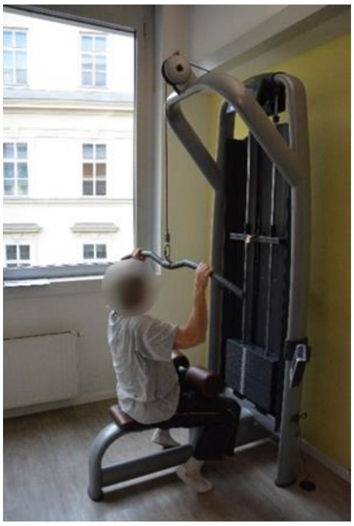 |

| Lower body | |
|---|---|
| M. quadriceps femoris | **<u>C1: Leg Press</u>**<br>*Starting position: sitting on the chair of the machine, lower back touching the back of the chair; feet are positioned about shoulder width on the plate, toes looking up or slightly outwards; knees are bend as far as a comfortable condition is given; hands are holding the handles on the side*<br>o Pressing the plate forward until the knees are almost stretched, but not to a full extent<br>o Return to the starting position<br>o Exhale while pushing the weight forward; inhale while moving the weight back<br>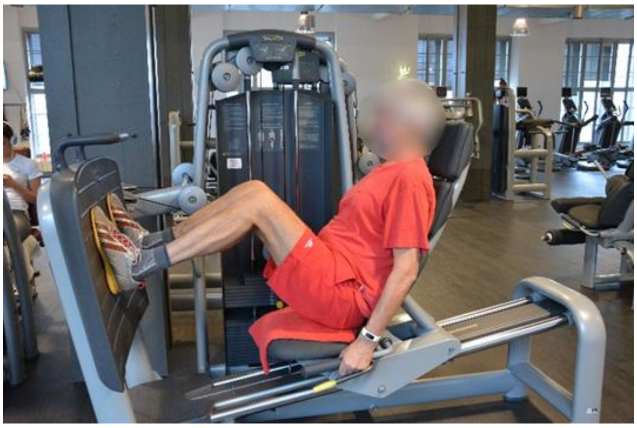 |

| Core | |
|---|---|
| M. erector spinae, M. gluteus maximus | **<u>C2: Front Plank with alternating leg raise</u>**<br>*Starting position: forearm braced on the bench, while the upper and lower body build a straight line, with the toes touching the ground; neck is in a neutral position*<br>o Legs get raised in an alternating sequence and hold shortly in the air<br>o Intensity can be increased by lowering the position (bench to ground)<br>o If using the forearms is too heavy, just the hands can be braced and a push up position can be taken in<br>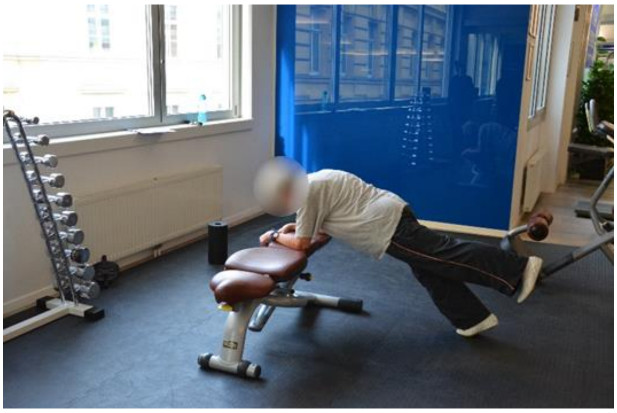 |

| Upper body | |
|---|---|
| M. latissimus dorsi, M. teres major, M. rhomboideus major, M. deltoideus posterior, M. brachioradialis | **D1: Rowing**<br>*Starting position: sitting with a straight back on the bench; sternum touching the pad; elbows are extended and hands are holding the handles*<br>○ Pull the weights to your body until the handles are reaching the chest<br>○ Move the weight controlled back to the starting position<br>○ Exhale while pulling the weight to your body; inhale while moving the weight back<br>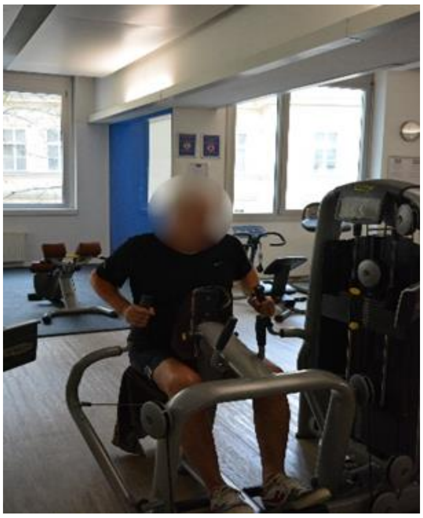 |
| M. pectoralis major, M. triceps brachii, M. deltoideus anterior | **D2: Chest Press**<br>*Starting position: sitting on the bench with the back straight; elbows are bend and hands holding the bar in neutral position*<br>○ Press the weight forward until the elbows are almost extended<br>○ Let the weight slightly move back<br>○ Exhale while pushing the weight forward; inhale while moving the weight in the starting position<br>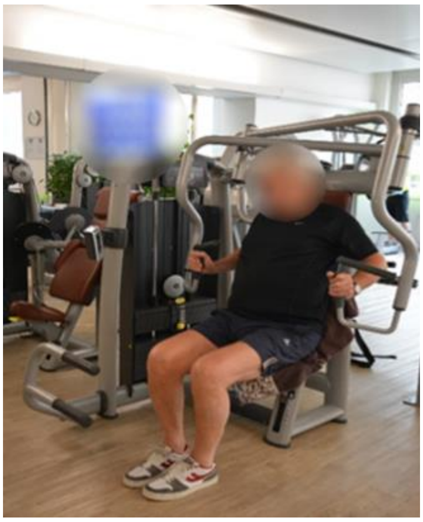 |

* Involved muscles from: Delavier, F. (2006). Strength training anatomy. Champaign, IL: Human Kinetics.
